# Flexible decision-making relative to reward quality and tool functionality in Goffin cockatoos (*Cacatua goffiniana*)

**DOI:** 10.1038/srep28380

**Published:** 2016-06-23

**Authors:** I. B. Laumer, T. Bugnyar, A. M. I. Auersperg

**Affiliations:** 1 Department of Cognitive Biology, University of Vienna, Althanstr. 14, 1090, Vienna, Austria; 2 Comparative Cognition, Messerli Research Institute, University of Veterinary Medicine, Veterinärplatz 1, 1210, Vienna, Medical University of Vienna, University of Vienna, Vienna, Austria

## Abstract

Decisions involving the use of tools may require an agent to consider more levels of relational complexity than merely deciding between an immediate and a delayed option. Using a new experimental approach featuring two different types of tools, two apparatuses as well as two different types of reward, we investigated the Goffin cockatoos’ ability to make flexible and profitable decisions within five different setups. Paralleling previous results in primates, most birds overcame immediate drives in favor of future gains; some did so even if tool use involved additional work effort. Furthermore, at the group level subjects maximized their profit by simultaneously considering both the quality of an immediate versus a delayed food reward (accessible with a tool) and the functionality of the available tool. As their performance levels remained stable across trials in all testing setups, this was unlikely the result of a learning effect. The Goffin cockatoos’ ability to focus on relevant information was constrained when all task components (both food qualities, both apparatuses and both tools) were presented at the same time.

The ability to flexibly decide whether or not to sacrifice an immediate option in the prospect of a more profitable option is likely associated to high levels of delay tolerance and cognitive plasticity. So far, among non-human animals, there is evidence that some primates, dogs, corvids and parrots can overcome immediate drives for more than a couple of seconds in order to maximize their future profits and can flexibly assess costs and benefits of an action before executing it (e.g. refs 1, 2, 3, 4, 5, 6, 7, 8, 9, 10).

Nevertheless, in decision-making processes in natural situations there are often more levels of relational complexity involved than merely deciding between an immediate and a delayed option. In order to maximize profit an agent may, for example, need to take into account the predictability (e.g. refs 11, 12, 13, 14), the reachability, as well as the presence and/or the functionality of available means to obtain a goal. In natural contexts, all these decisions may also need to be traded off against an immediately available, but less preferred option. Despite the substantial number of studies on animal decision-making, only a few primate studies include tool use in their task design: Tufted capuchin monkeys (*Sapajus spp.*) were able to delay eating a rod-shaped food (celery or pretzel) in order to use it as a tool to get access to a more preferred food (peanut butter in a tube). When the respective apparatus was not baited, all subjects immediately ate the initial rod-shaped food item^15^. Correspondingly, in an exchange task tufted capuchins flexibly traded a food item for a tool solely if the tool could be used for obtaining food of higher quality^16^. Chimpanzees (*Pan troglodytes*) and an orangutan (*Pongo abelii*) chose a tool over an immediate preferred food reward (grape) to use it 70 minutes later for obtaining a more preferred fruit soup^17^.

The latter tasks designs have been limited to tool using primates. As yet, therefore, it is unclear whether similar abilities could have evolved in other species, or whether the cognitive link between tool use and self control is an adaptive specialization to tool use. Corvids and parrots may represent promising candidate species as they seem to possess high levels of impulse control in delay of gratification tasks^2^,^5^,^7^ and some species have shown the capacity for flexible tool use (e.g. refs 18, 19, 20, 21). Goffin cockatoos may represent an appropriate parrot model for investigating such tool related decision-making. They are feeding generalists^22^ and show high levels of cognitive plasticity in a number of different problem solving tasks^18^,^19^,^23^,^24^. Furthermore, Goffin cockatoos tested in an exchange task were able to control an immediate impulse for up to 80 seconds to maximize a qualitative payoff. There was also evidence for plasticity in their decision-making, as subjects exchanged significantly more often for their most preferred food than for their second preferred food^2^. In contrast to species such as the New Caledonian crow^25^, Goffin cockatoos do not depend on tool-obtained food and are unlikely to be habitual tool users in the wild (recent field observations did not show any evidence of object insertions; unpublished data). However they have repeatedly shown complex forms of tool use and tool manufacture under laboratory conditions^18^,^19^.

This study investigates the flexibility of decision-making in Goffin cockatoos using a series of tool selection tasks. Specifically, we give birds binary choice tests and examine whether they can discriminate between the quality of an immediate available food reward and a food reward that can only be accessed with a tool (similar to primates^15^,^16^,^17^, while also taking into account the functionality of the available tool. We use a new experimental approach featuring two different types of tools (stick and ball, see methods) and their respective apparatuses (stick- and tube-apparatus, see methods) as well as two highly desired, although differently preferred, food rewards of different qualities. Specifically, we want to examine to what degree subjects can make binary decisions i) relative to the difference in quality of an immediate food reward (located outside of the apparatus) versus a food reward located inside of the apparatus and the presence of a tool (as shown in primates^15^,^16^,^17^) and ii) relative to the functionality of the available tool in the context at hand.

We set up five tests (*Tool selection test* (*TST*), *Motivation test* (*MT*), *Quality allocation test* (*QAT*), *Tool functionality test* (*TFT*) and *Tool selection/quality allocation test* (*TSQAT*); for detailed description see methods) in order to dissociate several aspects of tool related decision-making. These include tool selection, flexible value attribution to food rewards and tools, work effort, individual motivation, and the ability to focus on relevant task affordances to maximize the outcome (see methods). We hypothesize that the value of a tool relative to a food item should change depending upon its utility: the optimal strategy would be to only choose a tool over an immediate food reward if the present apparatus is accessible with the available tool and if the difference in quality between the immediate food item and the food item inside the apparatus is sufficiently high to compensate the work effort. Optimizing subjects should not choose the tool over the immediate food reward in controls in which the tool is non- functional or when the immediate food is of the same or of higher quality as the reward offered inside the apparatus. Furthermore, when confronted with more than one apparatuses, the subject should choose the tool functional for the apparatus containing the food of the highest quality. Given that Goffin cockatoos are able to accomplish tool use and to inhibit eating a preferred food item over extended delays to exchange it for a food of higher quality^2^,^18^,^19^, we expected them to choose a tool over a low quality immediate reward to obtain a high quality reward. We had no *a priori* expectation on whether or not the birds could handle the relational complexity of making profitable decisions also in regard to the functionality of the available tool.

## Results

The GLMM for the pretest (*tool selection test, TST)* showed a significant effect for ‘Group’ (after being tested in the *TST*, subjects were split up into two groups; each group received the following tests in a different order; for details see methods; F = 7.993, df1 = 1, df2 = 20, p = 0.01, Coefficient = 1.375, SE = 0.922). The GLMM for the quality allocation test (QAT) showed a significant effect for the ‘location of the most preferred food item’ (inside vs. outside the apparatus; see methods for details; F = 4.868, df1 = 1, df2 = 47, p = 0.036). No other effects were found in the remaining GLMMs for the following tests (for details of the GLMMs see methods; for detailed statistical results see SI, section A, Table S1).

### Pretest: Tool selection test (TST)

When the stick apparatus alongside both tools was present, all birds chose the correct tool significantly above chance level (one sample Wilcoxon Test, Group A: T^+^ = 21; p^exact^ = 0.031, n = 6; Group B: T^+^ = 26, p^exact^ = 0.047, n = 7; see Fig. 1.1). Birds of Group A showed a non-significant tendency to choose the correct tool more often in the condition in which the stick apparatus was present than birds of Group B (Mann-Whitney U Test, U = 8, p = 0.074, n^GroupA^ = 6, n^GroupB^ = 7). In the tube apparatus condition, there was no difference between the groups (Mann-Whitney U Test, U = 13, p = 0.280, n^GroupA^ = 6, n^GroupB^ = 7). Subjects chose the ball tool significantly more than expected by chance when the tube apparatus was present (one sample Wilcoxon test; T^+^ = 90, p^exact^ < 0.001, n = 13; see Fig. 1.1). There was no significant difference between the birds’ choice in the stick- and the tube-apparatus condition (paired Wilcoxon test; T^+^ = 52, p^exact^ = 0.685, n = 13). Nevertheless, there was a learning effect when the stick apparatus was present: subjects correctly chose the stick significantly more often in the last six trials than in the first six trials (paired Wilcoxon test; T^+^ = 79, p^exact^ = 0.017, n = 13). This was the only condition throughout the entire experiment for which we detected a learning effect (for detailed statistical results see SI, section A, Table S2).

### Motivation test (MT)

As expected from an optimizing agent (avoiding work effort), subjects chose the immediate food more often than the tool when the food types outside and inside the apparatus were of the same quality (subject’s most preferred food MPF; see methods below; one sample Wilcoxon test; stick-apparatus: T^+^ = 76, p^exact^ = 0.033, n = 13; tube-apparatus: T^+^ = 88, p^exact^ = 0.001, n = 13; see Fig. 1.2). Subjects’ choices did not differ between the stick- or tube-apparatus conditions (Paired Wilcoxon test; T^+^ = 64, p^exact^ = 0.216, n = 13).

### Quality allocation test (QAT)

As expected from an optimizing agent, subjects chose the functional tool over their third preferred food (TPF, see methods), when the most preferred food (MPF) was located inside the apparatus, (One sample Wilcoxon test; stick-apparatus: T^+^ = 81, p^exact^ = 0.010, n = 13; tube-apparatus: T^+^ = 89, p^exact^ < 0.001, n = 13; see Fig. 1.3). Specifically when the TPF was located inside the tube-apparatus, subjects chose the MPF over the tool above chance expectation (One sample Wilcoxon test; T^+^ = 79, p^exact^ = 0.017, n = 13). In the stick-apparatus condition, subjects tended to choose their MPF over the tool (One sample Wilcoxon test; T^+^ = 72, p^exact^ = 0.068, n = 13). There was a non-significant trend to making more correct choices when the MPF was inside rather than outside the apparatus in the stick-apparatus condition (Paired Wilcoxon test; T^+^ = 70, p^exact^ = 0.080, n = 13) but not in the ball-apparatus condition (Paired Wilcoxon test; T^+^ = 59, p^exact^ = 0.376, n = 13).

### Tool functionality test (TFT)

As expected from an optimizing agent, subjects chose the tool over the TPF when the tool was functional (One sample Wilcoxon test; stick-apparatus: T^+^ = 81, p^exact^ = 0.010, n = 13; tube-apparatus: T^+^ = 84, p^exact^ = 0.005, n = 13). In contrast, subjects chose the TPF over the tool when the tool was non-functional (One sample Wilcoxon test; stick-apparatus: T^+^ = 85, p^exact^ = 0.003, n = 13; tube-apparatus: T^+^ = 85, p^exact^ = 0.003, n = 13; see Fig. 1.4).

### Tool selection/quality allocation test (TSQAT)

In contrast to the other tests, subjects did not optimize their payoff above chance expectation when both apparatuses as well as both food qualities were present and they could choose one of both tool types (One sample Wilcoxon test; stick-apparatus: T^+^ = 47, p^exact^ = 0.946, n = 13; tube-apparatus: T^+^ = 52, p^exact^ = 0.685, n = 13; see Fig. 1.5). The subjects’ decision was not based on a side preference (Chi-square test; for all birds p > 0.05; for details see Table S3 in SI, section B). However, seven subjects showed a tool preference (Chi-square test; all p < 0.01), with three birds preferring the ball over the stick and four birds preferring the stick over the ball (see Table S4 in SI, section B). Further, after tool selection, 12 out of 13 subjects operated the apparatus for which their preferred tool was functional significantly above chance expectation (see Table S5 in SI, section B).

### Individual Performance

In each of the previous testing conditions except the *TSQAT,* some individuals performed spontaneously above or close to chance expectation (see SI section C for detailed individual results).

Five birds chose the profitable option in the stick- and/or tube-apparatus conditions in the *MT* above chance level (see SI, section C, Table S7). In the *QAT*, five subjects instantly chose the more profitable option above chance level in both conditions of the stick- and/or tube-apparatus. Three more birds tended to do so (SI, section C, Table S8). In the *TFT*, three birds significantly chose the profitable option in both test conditions of the stick- and/or tube-apparatus (SI, section C, Table S9). One more bird tended to do so. Goffin cockatoo “Muppet” instantly passed all conditions of the ball- and/or stick-apparatus condition of the *MT*, *QAT* and *TFT*. Based on performance in the *TFT* and/or the *QAT*, alternative learning strategies are plausible for a few subjects, but unlikely for the majority of individuals (see SI, section D).

## Discussion

After being trained to use two types of tools, Goffin cockatoos were able to attribute each tool to its respective apparatus once given a choice in a tool selection test. Like habitually tool-using primates^15^,^16^,^17^, most cockatoos did overcome immediate drives in favor of future gains, some even when this implied tool use as a work effort. Critically, at group level, subjects also succeeded in all tasks involving different levels of relational complexity. They could: i) Simultaneously consider the presence of an available tool as well as the difference in food quality between the immediate food and the food inside the apparatus; ii) at the same time attend to the functionality of the available tool in that context. As their performance levels did not improve across trials in the respective tests (*QAT*, *MT, TFT*), this was unlikely the result of learning. The cockatoos’ failure in the last test (*TSQAT*), in which six dimensions had to be considered simultaneously, indicates possible limitations in their information processing.

In the *MT* both options, choosing the tool as well as accessing the immediate food item, led to the same payoff. Here, subjects chose the food over the tool above chance expectation on the group level and some on the individual level. Goffin cockatoos show strong tendencies for complex, unrewarded object manipulation in- and outside of the foraging context^24^,^26^. Nevertheless, using a tool in the experiment seems to be judged as an additional work effort by some subjects and is therefore included in the decision-making process. In contrast, when the respective tool led to a higher payoff than the immediately available one (*QAT*), the effort for using a tool seemed to be compensated: On group level, subjects chose the tool significantly more often than the immediate food reward. They rarely selected the tool in control trials, in which the immediate food item was of higher quality than the reward located inside the apparatus. The previous results are in line with findings on habitually tool using primates (see introduction^15^,^16^,^17^): Tufted capuchin monkeys, one orangutan and chimpanzees were able to inhibit eating an immediate food reward if a more preferred food reward could be obtained by using/choosing a tool (although different task designs were employed). Beyond this, Goffin cockatoos seem to have the capacity to not only to consider the difference in food quality but also the functionality of the available tool (*TFT*). Subjects chose the immediate, lower quality food item over the tool when the offered tool was non-functional but chose the tool over the immediate lower quality food when the respective tool was functional. To our knowledge, such a task design has not yet been applied to primates.

Nevertheless, the Goffins’ group results need to be regarded with some caution: On an individual level, birds chose the correct option either above or close to conventional significance levels in some but not all of the testing conditions. However, a few subjects (notably the male Muppet and the female Moneypenny) continuously showed high performance levels in nearly all conditions of the two tests. Although individual performance is not always consistent, in some animals alternative individual strategies seemed apparent (see SI section D for a more detailed description). For example subject “Figaro” chose the non-functional ball tool over the TPF when confronted with the stick-apparatus in his first trial of the *TFT*. During the time interval before the next trial, he manufactured a tool of the required length out of the armrest of the chair (Figaro had made tools before out of wooden blocks, see ref. 18) and successfully retrieved the reward (see Movie S2, SI section E). In the following trials, Figaro often continued to choose the non-functional tool over the TPF and attempted to manufacture the adequate tool in the time between trials to access his MPF located inside the apparatus.

In the final task (*TSQAT*), subjects seemed unable to focus their attention on all relevant cues at the same time and subsequently could not maximize their profit when both apparatuses, both food qualities as well as both tools were present simultaneously. In this task, subjects seemed to limit their attention to the functionality of their preferred tool rather than to the difference in food quality of the apparatuses’ contents: they typically operated the apparatus for which their preferred tool was functional, even though it contained the low quality food in half of the occasions. Since they were given 30 seconds prior to the decision-making event, we believe we can rule out that this was due to insufficient inspection time. Relational complexity increases with the number of dimensions that must be considered simultaneously^27^ (*QAT/TFT: one apparatus, one tool, two food types; TSQAT:* two apparatuses, two tools, two food types). It seems possible that subjects faced some overload/limits: A plausible limit could be the cognitive capacity to flexibly recombine all relevant information when the task at hand required considering more than four factors at the same time. Nevertheless, the animals’ failure of the TSQAT could also be due to perceptual, attentional, or working memory constraints, or a combination of some of these factors^28^.

Overall, the results of this study illustrate that tool related decision-making relative to a gain in quality is neither limited to primates nor to adaptive specialists but can arise from relatively general modes of cognitive processing. In this case, these are most likely a combination of high behavioural flexibility, sensorimotor and impulse control. Goffin cockatoos seem to be able to incorporate the functionality of the tool at hand as an additional component into their decision-making process. Within this study, the Goffins did however, hit a limit in terms of working memory capacity with subjects being unable to consider a total of six task components simultaneously. As tool related decision-making beyond selection tasks (e.g. refs 20, 29, 30, 31) has not yet been addressed in birds and other non-primates, our understanding of the mechanisms involved in this type of multidimensional decision-making is still in its infancy. It would represent an important next step to probe its limits systematically in a broader range of species, for example incorporating specialist tool-makers such as New Caledonian crows.

## Methods

### Subjects & housing

We tested 13 adult Goffin cockatoos, five females and eight males (hatched between 2007 and 2011; see SI, Table S11, Section F). All subjects were hand-raised and are permanently kept in a large, enriched aviary (indoors: 45 m^2^ ground space, 3–6 m high wall to gable; outdoors: 150 m^2^ ground space, 3–4.5 m high). Food consisting of a selection of fresh fruits, seeds, various protein sources, minerals and fresh drinking water were available *ad libitum*. The indoor area was kept above 20 °C from October through May.

All animals had CITES certificates and were registered at the district’s administrative animal welfare bureau (Bezirkshauptmannschaft St. Pölten Schmiedgasse 4–6, A-3100; St. Pölten, Austria). These housing conditions comply with the Austrian Federal Act on the Protection of Animals (Animal Protection Act –§ 24 Abs. 1 Z 1 and 2; § 25 Abs. 3 – TSchG, BGBl. I Nr. 118/2004 Art. 2). As all experiments were appetitive, non-invasive and based exclusively on behavioural tests, they are not classified as animal experiments under the Austrian Animal Experiments Act (§ 2. Federal Law Gazette No. 501/1989). All birds were marked with coloured leg bands for identification.

All birds had experience with individual testing in problem solving tasks: a sequential lock problem^24^, Piagetian object permanence^23^ and a food exchange task (2, see introduction). Most subjects had experience inserting compact tools into a vertical tube (unpublished data) and four subjects (Figaro, Kiwi, Dolittle & Pipin) had experience retrieving a reward from behind a wire mesh using a stick tool^18^,^19^.

### Apparatus

**Figure 2:**
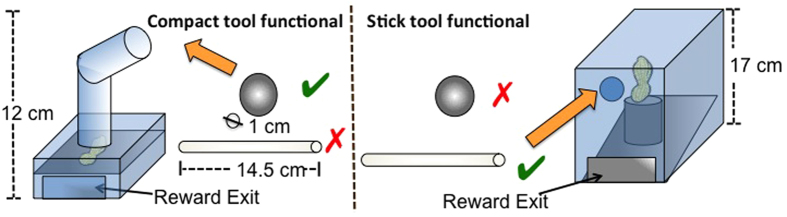
Left: The tube apparatus can only be operated by inserting a ball-shaped tool. The reward rests on a collapsible platform. Right: The stick apparatus can only be operated by inserting the stick through the upper hole and by poking the reward off a wooden platform.

Prior to testing, all subject received training with the two individual apparatuses used in this experiment (see Supplementary Information SI, section G for details). Before testing started, all birds could reliably retrieve food from each apparatus when the respective tool was present. We used two different apparatuses (tube & stick) and two types of tools (ball & stick) in this experiment. The tube-apparatus was a transparent food box with a vertical tube on top. The top bit of the tube was slanted, hindering the insertion of a stick-type tool (see Fig. 2). The reward rested on a collapsible platform inside the box. When a ball-shaped tool was inserted at the top opening of the tube, the platform collapsed and the food was released to the subject at the lower end of the box. The stick apparatus was a transparent rectangular box with a central opening (ca. 1.3 cm). The food reward rested on a small platform on a slanted plate inside the box. The reward could be poked off the platform by inserting a stick through the central opening and was released to the subject at the lower end of the box (see Fig. 2).

### Food preference tests

The three most preferred food rewards (always fully eaten) out of an original choice of 20 different food types were selected. Prior to testing, subjects received a food quality preference test in which relative preferences for the three foods were identified. To ensure that all foods were desirable but with a clear distinction in preference, we picked the most preferred and the third preferred out of the three for testing. To control for preference changes, food preferences were retested three times during the testing phase and, if necessary, an additional desirable food type was added to confirm that the most preferred food (MPF) was chosen over the third preferred food (TPF) in a minimum of 80% of binary choices. Subjects received the selected food types for the entire duration of the data collection only in test conditions (see SI section H for details).

### Testing set-ups and procedure

**Figure 3:**
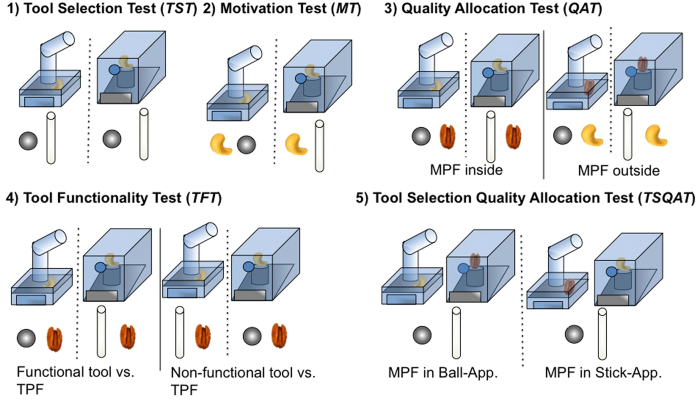
The conditions within each testing setup. (**1**) ***TST:*** both tools are present and MPF (most preferred food) is inside; (**2**) ***MT***: functional tool is present, MPF is inside and outside; (**3**) ***QAT***: functional tool is present; *left:* MPF is inside (depending on the condition either the tube or stick apparatus is present), TPF (third preferred food) is outside; r*ight*: MPF is outside, TPF is inside; (**4**) ***TFT:*** MPF is inside and TPF is outside; *left:* functional tool is present; *right:* non-functional tool is present; (**5**) ***TSQAT:*** both tools and both apparatuses are present; *left*: MPF is in tube apparatus; *right:* MPF is in stick apparatus.

All subjects participated in five tests (Fig. 3). The tests were conducted in two different orders to control for the possibility that the order of the tests might influence subjects’ performances in the subsequent tests. Prior to testing, the cockatoos were randomly divided into two equal groups (see SI, section F, Table S11). Group A first completed the Tool Selection Test (*TST*), then the Quality Allocation Test (*QAT*), the Motivation Test (*MT*), the Tool Functionality Test (*TFT*) and finally the Tool Selection/Quality Allocation Test (*TSQAT*). Group B also started with the *TST*, but then completed the *MT*, the *TFT*, the *QAT* and the *TSQAT*. Within each test, the conditions were presented in a semi-randomized order across sessions.

### Pretest: Tool selection test (TST)

As a critical first test, the *TST* investigated whether the cockatoos could select the functional tool. There were two possible conditions: either the stick- or the tube-apparatus (semi-randomized order across sessions), baited with subjects’ MPF, was placed on the experimental table (see Fig. 3.1, see Movie S1 in SI section E). Subjects received a minimum of two sessions of 12 trials in total in which they had to choose between the stick- and the ball-tool. As success in the TST was a precondition to enter further testing, it continued until subjects reached criterion (at least 83% of correct decisions in two consecutive sessions).

### Motivation test (MT)

The *MT* tested whether subjects had acquired a preference for the immediate food or for the food inside the apparatus. The subjects’ most preferred food (MPF) was located outside as well as inside the apparatus. The tool was always functional. There were two possible conditions: either the stick- or the tube-apparatus was placed on the experimental table (see Fig. 3.2; see Movie S1 in SI section E). Both conditions were presented in a semi- randomized order across sessions. Subjects received a total of two sessions of 12 trials.

### Quality allocation test (QAT)

The *QAT* tested whether subjects could increase profit by choosing a tool over an immediate food reward depending on the location of the higher quality food. One food reward was located inside, the other food reward outside the apparatus. The apparatus contained either the subjects’ MPF or TPF (in 50% of trials, semi-randomly balanced). The available tool was always functional (stick- or ball-tool depending on the apparatus present). There were four possible conditions that were presented in a semi- randomized order across sessions (see Fig. 3.3, see Movie S1 in SI section E). The *QAT* comprised four sessions of 12 trials.

### Tool functionality test (TFT)

The *TFT* tested whether subjects could correctly assess the functionality of a tool to increase profit. Subjects faced binary decisions between the TPF and either a functional or non-functional tool (in 50% of trials; semi-randomly balanced). The apparatus always contained the subjects’ MPF. There were four possible conditions that were presented in a semi- randomized order across sessions (see Fig. 3.4, see Movie S1 in SI section E). Subjects received a total of four sessions of 12 trials.

### Tool selection/quality allocation test (TSQAT)

The *TSQAT* tested if subjects could maximize their profit when all task components were present. Both apparatuses were placed on the experimental table containing food of different qualities (TPF and MPF). There were two possible conditions in which the MPF was either located inside the stick- or tube-apparatus (see Fig. 3.5, see Movie S1 in SI section E). The conditions were presented in a semi- randomized order across sessions. The *TSQAT* comprised two sessions of 12 trials.

### Testing procedure

During testing, the experimenter was standing at the experimental table while the bird was sitting opposite on the backrest of a chair (starting position). One of the two apparatuses was placed in centre of the table (semi-randomly balanced across sessions for each testing condition). The subject was asked to step up on the experimenter’s hand and was briefly lifted towards the apparatus to give it the opportunity to investigate its content (depending on the test it contained either the MPF or the TPF; see Movie S1 in SI section E). Thereafter, the bird was placed back into the starting position from where it could clearly see the apparatus’s content. Depending on the condition, subjects faced binary choices either between two tools or a tool and a food item placed on the right and left side of the apparatus. For all conditions, the side of presentation was semi-randomly balanced across sessions. Thereafter, the experimenter signalled the subjects to wait for three seconds extending her arm over the centre of the apparatus with the palm facing towards the subject. Subsequently, the bird was allowed to leave its starting position. Once an option was touched, the other was immediately removed (see Movie S1 in SI section E). If subjects chose the non-functional tool, they had to wait in front of the apparatus for 30 seconds until the beginning of the next trial. During testing, the experimenter wore mirrored sunglasses, avoided any head or facial movements and was not speaking.

### Analysis

All data was HD video recorded (JVC HD Everio Camcorder GZ-HM30) and coded *in situ* as well as from the videos. We conducted a Generalized Linear Mixed Model (GLMM) in IBM SPSS for each test (*TST*, *MT*, *QAT*, *TFT*, *TSQAT*) with ‘Sex’, ‘Group (order in which tests were received)’ and ‘Type of apparatus (stick or tube)’ as fixed effect factors and ‘Subject’ as random effect factor. As response variable, we used the number of correct choices out of the total of 12 trials for each test. For *QAT,* we additionally looked at ‘MPF inside/outside apparatus’ as fixed effect; for *TFT* we additionally looked at ‘Tool functional/non-functional’. As the data did not meet the criteria for parametric analysis, we used non-parametric two-tailed statistics for further analysis. Statistical tests were conducted in IBM SPSS using exact procedures. To investigate a possible learning effect we additionally ran a pairwise comparison (paired Wilcoxon tests, p < 0.05) for the first and last six trials of each condition for each test. Although testing was carried out until criterion in the *TST*, only performance in the first two sessions was analysed.

## Additional Information

**How to cite this article**: Laumer, I. B. *et al*. Flexible decision-making relative to reward quality and tool functionality in Goffin cockatoos (*Cacatua goffiniana*). *Sci. Rep.* **6**, 28380; doi: 10.1038/srep28380 (2016).

## Supplementary Material

Supplementary Movie S1

Supplementary Movie S2

Supplementary Information

## Figures and Tables

**Figure 1:**
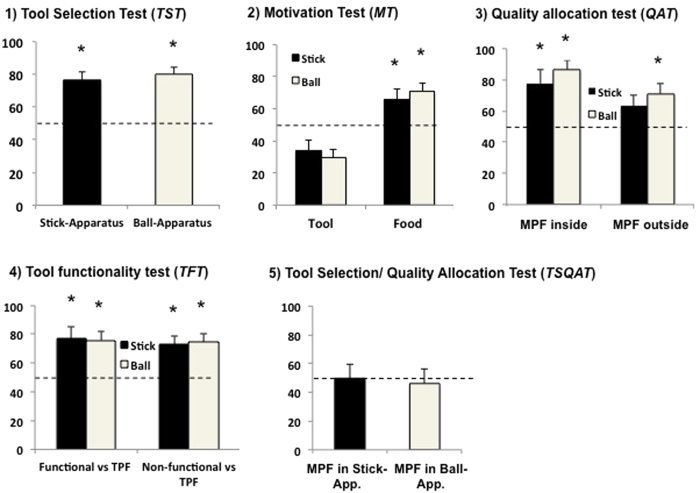
Mean percentages of correct/profitable choices for each condition within each test (**1**) *TST*, (**2**) *MT*, (**3**) *QAT,* (**4**) *TFT*, (**5**) *TSQAT*; n = 13). *p-value significantly above chance expectation. (MPF = most preferred food; TPF = third preferred food).
