# Supplementary material for: Flexible decision-making relative to reward quality and tool functionality in Goffin cockatoos (Cacatua goffiniana)
Source: Sci Rep. 2016 Jun 23;6:28380. doi: 10.1038/srep28380 (PMC4917853; doi:10.1038/srep28380)
Supplement: Supplementary Information [file srep28380-s3.doc]

**Title: Flexible decision-making** relative to reward quality and tool functionality

in Goffin cockatoos (*Cacatua goffiniana*)

**Authors:** Laumer, I. B., Bugnyar, T., Auersperg, A.M.I.

**Supplementary Information**

**A) Detailed statistical results of the GLMM analysis and the paired Wilcoxon tests (to investigate learning effects)**

**Table S1:** Results of the GLMM analysis for each test (n=13). P-values below 0.05 are highlighted in yellow.

|  | **Fixed effects** | **F** | **df1** | **df2** | **p** | **Coefficient** | **SE** |
| --- | --- | --- | --- | --- | --- | --- | --- |
| **TST** |  |  |  |  |  |  |  |
|  | Group | 7.312 | 1 | 22 | 0.013 | 1.844 | 0.682 |
|  | Sex | 1.201 | 1 | 22 | 0.285 | 0.765 | 0.699 |
|  | Type of apparatus | 1.201 | 1 | 22 | 0.502 | -0.462 | 0.677 |
|  |  |  |  |  |  |  |  |
| **MT** |  |  |  |  |  |  |  |
|  | Group | 0.003 | 1 | 22 | 0.959 | -0.063 | 1.208 |
|  | Sex | 2.862 | 1 | 22 | 0.105 | 2.094 | 1.238 |
|  | Type of apparatus | 0.819 | 1 | 22 | 0.375 | -0.538 | 0.595 |
|  |  |  |  |  |  |  |  |
| **QAT** | Group | 0.775 | 1 | 47 | 0.383 | 0.883 | 1.003 |
|  | Sex | 1.108 | 1 | 47 | 0.298 | 1.082 | 1.028 |
|  | Type of apparatus | 1.497 | 1 | 47 | 0.227 | -1.000 | 0.871 |
|  | MPF inside/outside of apparatus | 4.868 | 1 | 47 | 0.036 | 1,769 | 0.871 |
|  |  |  |  |  |  |  |  |
| **TFT** | Group | 0.044 | 1 | 47 | 0.834 | -0.172 | 0.815 |
|  | Sex | 0.046 | 1 | 47 | 0.831 | -0.18 | 0.836 |
|  | Type of apparatus | 0.000 | 1 | 47 | 1 | 0 |  |
|  | Tool functional/non-functional | 0.145 | 1 | 47 | 0.705 | 0.308 | 0.809 |
|  |  |  |  |  |  |  |  |
| **TSQAT** | Group | 0.033 | 1 | 22 | 0.875 | 0.312 | 1.714 |
|  | Sex | 0.053 | 1 | 22 | 0.819 | -0.406 | 1.757 |
|  | Type of apparatus | 0.074 | 1 | 22 | 0.789 | 0.462 | 1.701 |

We tried to reduce the model with Akaike criterion and found that the full model for the *TST*, *MT*, *QAT*, *TFT* and *TSQAT* had highest support.

**Table S2:** Results of the paired Wilcoxon tests for the first and last six trials of each condition for each test (n=13).

|  | T^+^ | p (exact Sig., 2-tailed) |
| --- | --- | --- |
| TST Stick-apparatus | 79 | 0.017 |
| TST Ball-apparatus | 66 | 0.168 |
| QAT Stick-app., MPF inside | 51 | 0.735 |
| QAT Stick-app., MPF outside | 73 | 0.057 |
| QAT Ball-app., MPF inside | 62 | 0.273 |
| QAT Ball-app., MPF outside | 59 | 0.376 |
| TFT Stick-app., tool functional | 65 | 0.191 |
| TFT Stick-app., tool non-functional | 68 | 0.127 |
| TFT Ball-app., tool functional | 70 | 0.080 |
| TFT Ball-app., tool non-functional | 63 | 0.244 |
| TSQAT, MPF in Stick-apparatus | 63 | 0.244 |
| TSQAT, MPF in Ball-apparatus | 50 | 0.787 |

**B) Side/Tool preferences and number of correct tool insertions in the *TSQAT***

**Table S3:** Results of Chi-square tests for each individual (Figaro-Heidi) of each Group (A & B) in the *TSQAT* for side-preferences.

| ***TSQAT: Sidepreference?*** | |  |  |  |  |  |
| --- | --- | --- | --- | --- | --- | --- |
| Name | Group | Left | Right | Chi-square | Exact Sig. | df |
| Figaro | A | 14 | 10 | 0.667 | 0.541 | 1 |
| Pipin | A | 11 | 13 | 0.167 | 0.839 | 1 |
| Kiwi | A | 14 | 10 | 0.667 | 0.541 | 1 |
| Zozo | A | 7 | 17 | 4.167 | 0.064 | 1 |
| Moneypenny | A | 14 | 10 | 0.667 | 0.541 | 1 |
| Olympia | A | 9 | 15 | 1.500 | 0.307 | 1 |
| Muki | B | 13 | 11 | 0.167 | 0.839 | 1 |
| Muppet | B | 11 | 13 | 0.167 | 0.839 | 1 |
| Konrad | B | 10 | 14 | 0.667 | 0.541 | 1 |
| Dolittle | B | 12 | 12 | 0.000 | 1.000 | 1 |
| Fini | B | 16 | 8 | 2.667 | 0.152 | 1 |
| Mayday | B | 13 | 11 | 0.167 | 0.839 | 1 |
| Heidi | B | 12 | 12 | 0.000 | 1.000 | 1 |

**Table S4** Results of Chi-square tests for each individual (Figaro-Heidi) of each Group (A & B) in the *TSQAT* for tool preferences. Results that are significantly above chance expectation are marked in yellow (p<0.01) and orange (if p<0.001) respectively.

|  | |  |  |  |  |  |
| --- | --- | --- | --- | --- | --- | --- |
| Name | Group | Stick | Ball | Chi-square | Exact Sig. | df |
| Figaro | A | 12 | 12 | 0.000 | 1.000 | 1 |
| Pipin | A | 5 | 19 | 8.167 | 0.007 | 1 |
| Kiwi | A | 22 | 2 | 16.667 | p<0.001 | 1 |
| Zozo | A | 7 | 17 | 4.167 | 0.064 | 1 |
| Moneypenny | A | 8 | 16 | 2.667 | 0.152 | 1 |
| Olympia | A | 15 | 9 | 1.500 | 0.307 | 1 |
| Muki | B | 23 | 1 | 20.167 | p<0.001 | 1 |
| Muppet | B | 21 | 3 | 13.500 | p<0.001 | 1 |
| Konrad | B | 2 | 22 | 16.667 | p<0.001 | 1 |
| Dolittle | B | 14 | 10 | 0.667 | 0.541 | 1 |
| Fini | B | 10 | 14 | 0.667 | 0.541 | 1 |
| Mayday | B | 23 | 1 | 20.167 | p<0.001 | 1 |
| Heidi | B | 0 | 24 | no value | p<0.001 | 1 |

**Table S5:** Number of correct tool-insertions, directly after tool selection, into the matching apparatus in the *TSQAT* (n=24). *= p<0.05 (18/24 correct), **= p<0.01 (19/24 correct); ***=p<0.001 (21/24 correct).

| Name | Insertion into correct apparatus (n=24 trails) |
| --- | --- |
| Muppet | 18* |
| Dolittle | 18* |
| Figaro | 19** |
| Muki | 21*** |
| Konrad | 18* |
| Pipin | 22*** |
| Zozo | 18* |
| Kiwi | 18* |
| Mayday | 19** |
| Moneypenny | 20** |
| Olympia | 19** |
| Fini | 16 |
| Heidi | 22*** |

**C) Individual Data for Tests**

**Table S6**: Number of correct trials out of a total of 12 trials for each condition of the *Tool selection test* (*TST*) for each individual (Figaro-Heidi) of each Group (A & B). The results show the performance in the first two sessions. Shaded areas indicate individual performance above chance level. *= p<0.05 (10/12 correct), **= p<0.01 (11/12 correct); ***=p<0.001 (12/12 correct).

|  |  | Tool Selection Test (TST) | | |
| --- | --- | --- | --- | --- |
|  |  |  |  |  |
|  |  | Session 1 + Session 2 | |  |
| Name | Group | Stick-App. (12 tr.) | Ball-App. (12 tr.) | Sessions until criterion |
| **Figaro** | A | 12*** | 11** | 2 |
| **Pipin** | A | 11** | 10* | 2 |
| **Kiwi** | A | 11** | 9 | 3 |
| **Zozo** | A | 10* | 9 | 3 |
| **Moneypenny** | A | 10* | 12*** | 2 |
| **Olympia** | A | 10* | 11** | 3 |
| **Muki** | B | 11** | 7 | 4 |
| **Muppet** | B | 6 | 12*** | 3 |
| **Konrad** | B | 11** | 8 | 3 |
| **Dolittle** | B | 8 | 10* | 3 |
| **Fini** | B | 7 | 10* | 4 |
| **Mayday** | B | 6 | 6 | 4 |
| **Heidi** | B | 7 | 10* | 3 |

**Table S7:** Number of profitable trials out of a total of 12 trials for each condition of the *Motivation test* (*MT*) for each individual (Figaro-Heidi) of each Group (A & B). Shaded areas indicate individual performance above chance level. *= p<0.05 (10/12 correct), **= p<0.01 (11/12 correct); ***=p<0.001 (12/12 correct).

|  |  | Motivation Test (MT) | | | |
| --- | --- | --- | --- | --- | --- |
|  |  | Session 1 + Session 2 | | | |
|  |  | Stick-Apparatus | | Ball-Apparatus | |
| Name | Group | Tool (12 Tr.) | Food (12Tr) | Tool (12 Tr) | Food (12 Tr) |
| **Figaro** | A | 0 | 12*** | 0 | 12*** |
| **Pipin** | A | 5 | 7 | 5 | 7 |
| **Kiwi** | A | 5 | 7 | 5 | 7 |
| **Zozo** | A | 6 | 6 | 2 | 10* |
| **Moneypenny** | A | 4 | 8 | 5 | 7 |
| **Olympia** | A | 1 | 11** | 6 | 6 |
| **Muki** | B | 0 | 12*** | 0 | 12*** |
| **Muppet** | B | 1 | 11** | 0 | 12*** |
| **Konrad** | B | 6 | 6 | 4 | 8 |
| **Dolittle** | B | 5 | 7 | 4 | 8 |
| **Fini** | B | 7 | 5 | 6 | 6 |
| **Mayday** | B | 8 | 4 | 5 | 7 |
| **Heidi** | B | 5 | 7 | 4 | 8 |

**Table S8:** Number of profitable trials out of a total of 12 trials for each condition of the *Quality allocation test* (*QAT*) for each individual (Figaro-Heidi) of each Group (A & B). Results of the birds that performed above chance level in both conditions of the stick- and/or ball-apparatus condition are marked in grey, slightly not significant performance (9/12 correct) is shaded in light grey. *= p<0.05 (10/12 correct), **= p<0.01 (11/12 correct); ***=p<0.001 (12/12 correct).

|  |  | Quality allocation Test (QAT) | | | |
| --- | --- | --- | --- | --- | --- |
|  |  | Session 1- Session 4 | | | |
|  |  | Stick-Apparatus | | Ball-Apparatus | |
| Name | Group | MPF inside (12 Trials) | MPF outside (12 Trials) | MPF inside (12 Trials) | MPF outside (12 Trials) |
| **Figaro** | A | 12*** | 7 | 12*** | 10* |
| **Pipin** | A | 12*** | 6 | 12*** | 9 |
| **Kiwi** | A | 7 | 10* | 9 | 9 |
| **Zozo** | A | 2 | 8 | 4 | 11** |
| **Moneypenny** | A | 9 | 8 | 12*** | 10* |
| **Olympia** | A | 4 | 8 | 6 | 8 |
| **Muki** | B | 12*** | 12*** | 12*** | 11** |
| **Muppet** | B | 3 | 11** | 10* | 10* |
| **Konrad** | B | 12*** | 9 | 11** | 8 |
| **Dolittle** | B | 12*** | 6 | 12*** | 8 |
| **Fini** | B | 12*** | 0 | 12*** | 1 |
| **Mayday** | B | 12*** | 5 | 11** | 5 |
| **Heidi** | B | 12*** | 9 | 12*** | 11** |

**Table S9:** Number of correct trials out of a total of 12 trials for each condition of the *Tool functionality test* (*TFT*) for each individual (Figaro-Heidi) of each Group (A & B). Results of the birds that performed above chance level in both conditions of the stick- and/ or ball-apparatus condition are marked in grey, slightly not significant performance (9/12 correct) is shaded in light grey. *= p<0.05 (10/12 correct), **= p<0.01 (11/12 correct); ***=p<0.001 (12/12 correct).

|  |  | Tool functionality Test (TFT) | | | |
| --- | --- | --- | --- | --- | --- |
|  |  | Session 1- Session 4 | | | |
|  |  | Stick-Apparatus | | Ball-Apparatus | |
| Name | Group | Functional vs TPF (12 Tr.) | Non-functional vs TPF (12Tr) | Functional vs TPF (12 Tr) | Non-functional vs TPF (12 Tr) |
| **Figaro** | A | 12*** | 6 | 11** | 7 |
| **Pipin** | A | 12*** | 11** | 11** | 11** |
| **Kiwi** | A | 6 | 10* | 7 | 10* |
| **Zozo** | A | 4 | 12*** | 4 | 11** |
| **Moneypenny** | A | 12*** | 9 | 9 | 10* |
| **Olympia** | A | 1 | 11** | 6 | 11** |
| **Muki** | B | 12*** | 6 | 7 | 11** |
| **Muppet** | B | 11** | 10* | 12*** | 11** |
| **Konrad** | B | 12*** | 7 | 11** | 4 |
| **Dolittle** | B | 6 | 10* | 6 | 9 |
| **Fini** | B | 10* | 8 | 11** | 8 |
| **Mayday** | B | 10* | 10* | 11** | 9 |
| **Heidi** | B | 12*** | 4 | 12*** | 4 |

**Table S10:** Number of profitable trials out of a total of 12 trials for each condition of the *Tool selection quality allocation test* (*TSQAT*) for each individual (Figaro-Heidi) of each Group (A & B). *= p<0.05 (10/12 correct), **= p<0.01 (11/12 correct); ***=p<0.001 (12/12 correct).

|  |  | Tool selection quality allocation Test (TSQAT) | |
| --- | --- | --- | --- |
|  |  |  |  |
|  |  | Session 1 + Session 2 | |
| Name | Group | MPF in stick-apparatus (12 trials) | MPF in ball-apparatus (12 trials) |
| **Figaro** | A | 4 | 4 |
| **Pipin** | A | 4 | 11** |
| **Kiwi** | A | 10* | 0 |
| **Zozo** | A | 3 | 8 |
| **Moneypenny** | A | 4 | 8 |
| **Olympia** | A | 9 | 6 |
| **Muki** | B | 12*** | 1 |
| **Muppet** | B | 11** | 2 |
| **Konrad** | B | 1 | 11** |
| **Dolittle** | B | 5 | 3 |
| **Fini** | B | 4 | 6 |
| **Mayday** | B | 11** | 0 |
| **Heidi** | B | 0 | 12*** |

**D) Information on individual strategies in the *QAT* and *TFT***

Note that, since only the minimum number of trials to reach statistical significance on an individual level was conducted for each test, motivational issues like “bad day” performances may have had a significant influence on individual data for a particular condition.

Nevertheless, some cases represent possible individual strategies in the *QAT* and/or *TFT*: In the *QAT*, “Fini” and “Mayday” preferred choosing the tool over the immediate food reward in nearly all or most trials, also if this implied loosing the MPF (see section D, Table S5 & S6). But in the TFT, “Mayday“ significantly chose the immediate food (TPF) over the tool when the tool was non-functional and chose the tool over the immediate TPF when the tool was functional in the stick-apparatus condition and tended to do so in the ball-apparatus condition (Table S6). Finally, subject „Fini“ chose the immediate TPF over the non-functional tool in 8/12 trials in both stick- and ball apparatus condition of the TFT (Table S6). Some subjects (e.g. Mayday, Fini) might have switched their strategy because the costs of making the unprofitable decision were higher in the TFT than in the QAT, since in the TFT choosing the non-functional tool led to no reward at all, whereas in the QAT the less profitable decision led to a piece of the third prefered food.

In contrast the behaviour of Goffin “Zozo” seems to denote scarce flexibility: “Zozo” chose the food reward located outside of the apparatus, independently of the reward quality in the *QAT* (Table S5). Zozo also tended to choose the immediate food reward over the tool in the following *TFT*, irrespectively whether the tool was functional or not (Table S6).

For some subjects it seems that the difference in reward-quality between the most and the third prefered food reward was bigger than for other subjects: For example in the *TFT*, “Heidi” often remained on the starting position for up to 30s only when the non-functional tool as well as the TPF was present. She thereafter frequently picked up the non-functional tool rather than the immediate food reward. When the functional tool was present, she immediately left the starting position in order to pick up the tool to access the MPF located inside of the apparatus in both stick- and ball- apparatus condition (see section D, Table S6). “Figaro” chose the non-functional ball tool over the TPF when confronted with the stick-apparatus in his first trial of the *TFT*. During the time interval before the next trial, he manufactured a functional tool with the required length out of the wooden chair (up to this point he had never made a tool out of a chair) and successfully retrieved the reward (see Movie S2). In the following trials Figaro often continued to choose the non-functional tool over the TPF and attempted to manufacture the adequate tool in the time between trials to access his MPF located inside of the apparatus (further tool manufactures of the chair could be prevented by removing it on time).

**E) Movie**

**Movie S1** Movie of the Tool selection test (*TST*), Motivation test (*MT*), Quality allocation test (*QAT*), Tool functionality test (*TFT*) and Tool selection quality allocation test (*TSQAT*).

**Movie S2** Movie of Goffin Figaro´s first trial of the TFT, in which he chose the non-functional tool over his third preferred food. In the interval between the next trial he manufactured a functional tool with the required length out of a wooden chair.

**F) Subject information**

**Table S11** Names, division in two testing groups, sex and age of the 13 Goffin cockatoos.

| Name | Group | Sex | Hatched |
| --- | --- | --- | --- |
| **Figaro** | A | male | 2007 |
| **Pipin** | A | male | 2008 |
| **Kiwi** | A | male | 2010 |
| **Zozo** | A | male | 2010 |
| **Moneypenny** | A | female | 2010 |
| **Olympia** | A | female | 2010 |
| **Muki** | B | male | 2011 |
| **Muppet** | B | male | 2010 |
| **Konrad** | B | male | 2010 |
| **Dolittle** | B | male | 2011 |
| **Fini** | B | female | 2007 |
| **Mayday** | B | female | 2011 |
| **Heidi** | B | female | 2010 |

At the time of testing (spring to autumn 2014) all individuals were adults.

**G) Tool training phase**

*1) Operating the apparatuses*

The tube apparatus could be operated by inserting a ball tool in order to obtain a food reward (food became available by collapsing a magnetic platform inside the apparatus). The stick apparatus could be operated with a stick tool. The reward was resting on a platform behind a Plexiglas **®** wall and was accessible by poking through a small hole in the wall.

*Results*

*Operating the stick-apparatus*

Three birds successfully inserted and operated the stick without any help within the first 5-session-blocks, with each session lasting for ten minutes (Figaro: 22min 24s, Dolittle: 26min 48s, Kiwi: 38min 55s). Two Goffins (Pipin, Mayday) failed in the first 5-session block, but successfully operated the stick-apparatus after observing a conspecific (three demonstrations by the conspecific while the subject was watching and afterwards ten minutes to try-out theirselves per session). Four subjects (Konrad, Moneypenny, Fini, Muki) succeeded within the next 5-session-block, in which the stick was 12 times per session pre-inserted by the experimenter (IBL) and the reward was poked off the platform by the subject, followed by a ten minutes block to try out by theirselves. The remaining four birds (Zozo, Muppet, Heidi, Olympia) successfully completed a stick insertion-shaping training after one to eight sessions. Once a bird was for the first time successful, the performance was displayed continuously.

*Operating the tube apparatus*

In a previous experiment (Auersperg et al., in prep.), four birds (Dolittle, Figaro, Fini, Moneypenny) inserted the ball into the tube-apparatus without any help within the first two sessions of 10 minutes. Pipin needed nine sessions to solve the task. Subjects that were not successful within 10 sessions, afterwards faced an apparatus with removed tube: the food reward could only be obtained by collapsing the platform by inserting the beak or foot. With that pre-experience, Mayday, Heidi (both after 21 sessions) and Zozo (23 sessions) managed to operate the original tube apparatus. Subjects that were not able to solve the task (Konrad, Muppet, Kiwi, Olympia) and subject Muki that did not participate in the previous study, received a ball insertion-shaping training prior to present study.

*2) Training phase*

Prior testing subjects received two training phases with the two apparatuses:

To give the birds the experience that each tool is a functional key to one but not to both apparatuses, subjects received five sessions of ten trials in which they were confronted with either the stick- or the ball-apparatus (semi-randomly mixed) and the respective non-functional tool. After two minutes the tool was replaced by the functional tool.

Thereafter, to ensure that subjects paid attention to the apparatuses´ food content, the Goffins received an unlimited number of sessions of 12 trials in which the apparatuses contained in random order in 25% the MPF, in 25% TPF and in 50% a useless stone. If the birds operated the apparatus although it contained the stone, they had to wait a duration of 30 seconds until the next trial. Birds were tested until they reached the criterion (showing one out of the following behaviors in a minimum of 88.8% in three consecutive sessions when confronted with the stone: subject refuses to insert the tool for at least ten seconds, discards the tool from the table or leaves the table).

**H) Quality preference test**

*Methods*

Subjects received a food quality preference test in which preferences for three desirable (always fully eaten) food items (cashew, pistachio, pecan nut; for one subject (Muki): pistachio, sunflower seeds, peanuts) were identified (eight sessions of 12 trials, so that all possible side and food combinations were tested 32 times). Two different, equally sized food items were placed simultaneously onto the experimental Table (1x1m) in front of the bird. Subjects were allowed to eat the food item first touched, the other food item was removed immediately. To control for possible preference changes, food preferences were retested three times during the testing phase and if necessary an additional desirable food type (sunflower seeds for three birds) was added to confirm that the most preferred food (MPF) was chosen over the third preferred food (TPF) in a minimum of 80% of binary choices. Subjects received the selected food types for the whole duration of the data collection only in test conditions.

*Results Quality preference test*

Subjects chose their respective MPF significantly more often than their TPF (Paired Wilcoxon test: T^+^=101, p^exact^<0.001, n=13; mean choice MPF over TPF: 95.89%; for details see Table S2).

**Table S12** Preference tests including all combinations (pi=pistachio; c=cashew nut; pe=pecan nut; s=sunflower seed, e=peanut; TPF= third preferred food, MPF= most preferred food). Note that for preference test 2 and 3, only the individual results for those birds that showed food preference changes are reported.

| ***Preference test 1*** | | | Combination 1 | | Combination 2 | | Combination 3 | | **Summary** | |
| --- | --- | --- | --- | --- | --- | --- | --- | --- | --- | --- |
| *Subjects* | *Hierarchy* | | *pecan* | *cashew* | *pistachio* | *cashew* | *pecan* | *pistachio* | **TPF** | **MPF** |
| *Dolittle* | pe<pi<c | | 9,4 | 90,6 | 18,8 | 81,3 | 21,9 | 78,1 | 9,4 | 90,6 |
| *Figaro* | pi<pe=c | | 50 | 50 | 0 | 100 | 100 | 0 | 0 | 100 |
| *Kiwi* | pi<pe<c | | 31,3 | 68,8 | 3,1 | 96,9 | 90,6 | 9,4 | 3,1 | 96,9 |
| *Konrad* | pi<pe<c | | 15,6 | 84,4 | 0 | 100 | 90,6 | 9,4 | 0 | 100 |
| *Muppet* | pi<pe<c | | 31,3 | 68,8 | 0 | 100 | 100 | 0 | 0 | 100 |
| *Pipin* | pi<pe<c | | 9,4 | 90,6 | 3,1 | 96,9 | 84,4 | 15,6 | 3,1 | 96,9 |
| *Zozo* | pi<c<pe | | 62,5 | 37,5 | 3,1 | 96,9 | 100 | 0 | 0 | 100 |
| *Fini* | pe<pi<c | | 9,4 | 90,6 | 18,8 | 81,3 | 25 | 75 | 9,4 | 90,6 |
| *Heidi* | pi<pe<c | | 3,1 | 96,9 | 0 | 100 | 100 | 0 | 0 | 100 |
| *Mayday* | pi<pe<c | | 12,5 | 87,5 | 9,4 | 90,6 | 59,4 | 40,6 | 9,4 | 90,6 |
| *Moneypenny* | pe<pi<c | | 0 | 100 | 6,3 | 93,8 | 9,4 | 90,6 | 0 | 100 |
| *Olympia* | pi<pe<c | | 0 | 100 | 0 | 100 | 81,3 | 18,8 | 0 | 100 |
|  |  | | *sunflower* | *peanut* | *pistachio* | *peanut* | *sunflower* | *pistachio* |  |  |
| *Muki* | pi<s<e | | 3,1 | 96,9 | 0 | 100 | 78,1 | 21,9 | 0 | 100 |
| ***Preference test 2*** | | | *pecan* | *cashew* | *pistachio* | *cashew* | *pecan* | *pistachio* |  |  |
| *Olympia* | | pe<pi<c | 3,1 | 96,9 | 25 | 75 | 31,25 | 68,75 | 3,1 | 96,9 |
| *Zozo* | | pi<pe<c | 43,75 | 56,25 | 6,25 | 93,75 | 84,4 | 15,6 | 6,25 | 93,75 |
|  | |  | *sunflower* | *cashew* | *pistachio* | *cashew* | *sunflower* | *pistachio* |  |  |
| *Mayday* | | s<pi< c | 15,6 | 84,4 | 21,9 | 78,1 | 6,2 | 93,8 | 15,6 | 84,4 |
| *Konrad* | | s<pi< c | 9,4 | 90,6 | 37,5 | 62,5 | 3,1 | 96,9 | 9,4 | 90,6 |
|  | |  | *sunflower* | *peanut* | *pistachio* | *peanut* | *sunflower* | *pistachio* |  |  |
| *Muki* | | s<e<pi | 15,6 | 84,4 | 100 | 0 | 0 | 100 | 0 | 100 |
| ***Preference test 3*** | | | *sunflower* | *cashew* |  |  |  |  |  |  |
| *Kiwi* | | s<c | 9,4 | 90,6 |  |  |  |  | 9,4 | 90,6 |
